# Association of *MIR17HG* and *MIR155HG* gene variants with steroid-induced osteonecrosis of the femoral head in the population of northern China

**DOI:** 10.1186/s13018-021-02669-y

**Published:** 2021-11-15

**Authors:** Tingting Liu, Yuju Cao, Changxu Han, Feimeng An, Tiantian Wang, Menghu Sun, Chao Ma, Qiumei Dong, Jianzhong Wang

**Affiliations:** 1 Inner Mongolia Medical University, Hohhot, 010110 Jinshan Development Zone China; 2 The Second Affiliated Hospital of Inner Mongolia Medical University, No. 1, Yingfang Road, Huhhot, 010030 Hui District China; 3 Zhengzhou Traditional Chinese Medicine (TCM) Traumatology Hospital, No.1266, First Street, Hanghai East Road, Zhengzhou, 450009 China; 4 Inner Mongolia Autonomous Region Hospital of Traditional Chinese Medicine, No. 11, Xingcheng District, Hohhott, 010010 China; 5 College of Traditional Chinese Medicine, Inner Mongolia Medical University, Hohhot, 010110 Jinshan Development Zone China

**Keywords:** Steroid-induced osteonecrosis of the femoral head, *MIR17HG*, *MIR155HG*, Single-nucleotide polymorphism, Case–control study

## Abstract

**Introduction:**

Steroid-induced osteonecrosis of the femoral head (ONFH) is a disease of the bone. Metabolism and genetic factors are generally considered to play an important role. The purpose of this study was to investigate the relationship between single-nucleotide polymorphisms (SNPs) in *MIR17HG* and *MIR155HG* and the risk of steroid-induced ONFH in the population of northern China.

**Methods:**

A total of 199 steroid-induced ONFH patients and 506 healthy controls were recruited for the study. Four SNPs of *MIR17HG* and seven SNPs of *MIR155HG* were genotyped by Sequenom MassARRAY. ORs and 95% CIs were used to evaluate the relationship between these SNPs and steroid-induced ONFH.

**Results:**

In the codominant model, patients with the *MIR17HG* SNPs (rs7318578) AA genotype had an increased risk of steroid-induced ONFH (OR = 1.79, *p* = 0.039); in the recessive model, patients with the *MIR17HG* SNP (rs7318578) AA genotype had an increased risk of steroid-induced ONFH (OR = 1.78, *p* = 0.032). Stratified analysis showed that a *MIR17HG* SNP (rs7318578) and the *MIR155HG* SNPs (rs77218221, rs11911469, rs34904192 and rs4143370) were closely related to different unornamented phenotypes of steroid-induced ONFH. Analysis of the clinical indicators revealed significant differences in high-density lipoprotein (*HDL-C*) levels between the ONFH group and the control group (*p* = 0.005). In the MIR17HG SNP (rs75267932), patients with different genotypes had different levels of triglyceride (*TG*). The MIR155HG SNPs (rs77699734, rs1893650, and rs34904192) showed differences in triglyceride (*TG*), high-density lipoprotein (*HDL-C*) and low-density lipoprotein (*LDL-C*) levels in patients with different genotypes.

**Conclusion:**

Our results confirm that *MIR17HG* and *MIR155HG* gene mutations are associated with steroid-induced ONFH susceptibility in the population of northern China, providing new evidence for the early detection and prevention of ONFH.

## Introduction

Osteonecrosis of the femoral head (ONFH), also known as avascularity necrosis of the femoral head, occurs mostly in young adults aged 30–50 and is a refractory disease in the Department of Orthopedics [23]. Due to hidden early symptoms, most patients with osteonecrosis develop to the middle and late stages when they are diagnosed with osteonecrosis, and there are varying degrees of femoral head collapse or loss of hip joint function, which seriously affect the quality of life of patients [30, 32, 36]. ONFH has different causes including steroids, alcohol, trauma, and idiopathic origins. Among them, steroid-induced ONFH accounts for the highest proportion and has become the main cause of femoral head necrosis [3]. A national epidemiological study on ONFH in Japan showed that steroid-induced ONFH accounted for 51% of the total cases [5]. Steroid-induced ONFH is a complex and multifactorial disease. A variety of internal and external factors jointly promote intramural microvascular lesions; thrombosis leads to insufficient blood supply and oxygen supply to the femoral head, and finally leads to bone cell death. However, the exact pathogenesis is still unclear. Clinical observations have found that not all patients treated with glucocorticoids have experienced femoral head necrosis, suggesting that there may be other risk factors or individual differences in the occurrence of glucocorticoid-induced ONFH. Some studies have shown that the difference between individuals may be related to gene polymorphism [18, 32].

Osteoblasts (OBs) and osteoclasts (OCs) are the main cells regulating bone homeostasis. Both cell types play a key role in bone tissue reconstruction, bone system integrity, mineral homeostasis in bone tissue, and bone metabolism balance [1, 13]. In the ONFH study, one of the pathogeneses was due to the imbalance of the OB/OC ratio and activity in bone tissue, which disrupted the dynamic balance between bone resorption and bone formation [21, 34].

MicroRNAs (miRNAs) are small, single-stranded, endogenous noncoding RNAs with a length of 21–25 nucleotides. They play biological roles by inhibiting the expression of target proteins at the posttranscriptional level [1, 29, 38]. According to the conformation analysis, more than one third of human genes are regulated by miRNAs, which indicates that miRNAs play an important role in regulating gene expression [10, 11]. *MiR-17* and *miR-155* are transcripts of *MIR17HG* and *MIR155HG*, respectively. *MiR-17* and *miR-155* are both multifunctional miRNAs, that play a consequential role in the occurrence, development, and prognosis of tumors [28, 38]. They also have important regulatory effects on bone metabolism. *MiR-17-92* is located on chromosome 13q31.3 of the host gene *MIR17HG* (also known as c13orf2 or mirage). Its expression is downregulated with the differentiation of OBs, and its expression is the lowest in mature OBs. These results indicate that *miR-17-92* regulates the proliferation, differentiation, and apoptosis of normal OBs [37]. In the study of OCs, it was found that *miR-155* inhibits osteoclast differentiation by mediating *IFN-b* [33].

Single-nucleotide polymorphisms (SNPs) are the most common type of genetic variation. At present, there is no study on the relationship between *MIR17HG* and *MIR155HG* SNPs and steroid-induced ONFH. In this study, we conducted a case–control study in a Chinese Han population to analyze the relationship between 11 SNPs and the risk of steroid-induced ONFH. Our results can be used as molecular markers for the diagnosis of osteonecrosis and provide a new theoretical basis for the early detection and prevention of osteonecrosis.

## Materials and methods

### Study participants

From 2018 to 2020, a total of 199 blood samples from steroid-induced ONFH patients were collected at Zhengzhou’s Traditional Chinese Medicine Hospital trauma center. The diagnosis of steroid-induced ONFH was defined as prednisolone with an average routine daily dose of 16.6 mg or a maximum daily common dose of 80 mg for at least 1 year [35]. All patients underwent anterior, posterior, and frog X-ray examinations. The diagnosis of ONFH was confirmed by MRI. All patients with steroid-induced ONFH had no history of chemotherapy or radiotherapy.

At the same time, 506 unrelated healthy people were recruited from the Zhengzhou Traditional Chinese Medicine Hospital as a control. All the subjects were of Han nationality and lived in Zhengzhou and its surrounding areas. Steroid-induced ONFH was excluded as follows: traumatic dislocation of the hip or other hip diseases, more than 400 ml of alcohol per week or having a clearly familial hereditary disease. In general, subjects with chronic diseases and diseases involving the brain, heart, liver, lungs, and other vital organs were excluded from the study.

The Ethical Committee of Zhengzhou Traditional Chinese Medicine Traumatology Hospital agreed to the study, and all participants signed written informed consent forms.

### Genotyping

Five-milliliter blood samples were collected in EDTA test tubes, centrifuged at 2000 rpm for 10 min, and stored at − 80 °C for future experiments. Genomic DNA was extracted by GoldMag extraction (GoldMag, China) and stored at 20 °C. The multichannel SNP quality extension detection method was developed using Agena MassARRAY Assay Design 4.0 software. The Agena MassARRAY system was used for SNP genotyping. Agena Bioscience Typer 4.0 software was used for data management and analysis. In addition, approximately 10% of the total samples were randomly selected for repeat genotyping, and the reproducibility was 100%.

### Statistical analysis

SPSS 20.0 software (SPSS, Chicago, IL, USA) was used for statistical analysis of the data. A chi-squared test was used to analyze whether the genotype distribution of the control group accorded with Hardy Weinberg’s equilibrium (HWE). In this study, *p* values less than 0.05 were statistically significant. We used a chi-squared test to calculate the distribution differences of alleles, genotypes, and haplotypes. We analyzed the relationship between genotypes of MIR17HG and MIR155HG polymorphisms and the risk of steroid-induced ONFH using different genetic models including codominant, dominant, recessive, and log-additive models; and calculated the odds ratios (ORs) and 95% confidence intervals (CIs) of logistic regression adjusted for age and sex. Then, stratified analysis was conducted according to age, gender, hip joint disease, and course of disease. Finally, linkage disequilibrium (LD) and haplotype structure were estimated by half software (version 4.2).

### Analysis of clinical indicators

Blood samples of the subjects were further analyzed. The Roche Cobas 8000-701 biochemical analyzer was used to detect blood lipid indexes in the body, and six biochemical indexes (TC, TG, HDL, LDL, ApoA1, ApoB). Two-tailed *T* test was used for quantitative variables, and chi-squared test was used for categorical variables. The expression of clinical indexes between the case group and the control group as well as different genotypes were analyzed. All *p* values in this study were two sided, and *p* value of less than 0.05 was the cutoff value for statistical significance.

### KEGG analysis of downstream genes regulated by MIR155HG

We searched out the downstream genes regulated by MIR155HG in (http://www.bio-bigdata.net/LncACTdb/index.html) and (http://mirtarbase.cuhk.edu.cn/) databases, using R software. KEGG analysis was performed on these genes.

## Result

Information on steroid-induced ONFH in patients and healthy individuals is shown in Table 1. A total of 199 patients (116 males and 83 females) were enrolled, with an average age of 41.21 ± 12.90 years; 506 healthy persons (423 males and 83 females) with an average age of 42.58 ± 13.15 years were included. Basic features included gender, age, hip lesions (unilateral or bilateral), and course ( > 25 months or ≤ 25 months). Steroid-induced ONFH patients were matched with the age of the control group (*p* = 0.212).

**Table 1:** Characteristics of the individuals in controls and steroid-induced ONFH patients

Variables	Cases (*n* = 199)	Controls (*n* = 506)	*p*
	*N* (%)	*N* (%)	
Age, years			
Mean ± SD	41.21 ± 12.90	42.58 ± 13.15	0.212^a^
≤ 45	129 (65%)	275 (54%)	
> 45	70 (35%)	231 (46%)	
Gender			< 0.001^b^
Male	116 (58%)	423 (84%)	
Female	83 (42%)	83 (16%)	
Hip lesions			
Unilateral	142 (71%)		
Bilateral	55 (28%)		
Course, months			
> 25	67 (34%)		
≤ 25	132 (66%)		

*P* < 0.05 indicates statistical significance.

^a^Independent sample *t* test.

^b^Two-sided chi-squared test.

The basic information of 11 SNPs in *MIR17HG* and *MIR155HG*, including gene, chromosome, location, allele, MAF, etc., is shown in Table 2. All these SNPs were in Hardy Weinberg’s equilibrium in the control group. The *Χ*^2^ test was used to calculate the difference in the allele frequency distribution between the case and the control group. We did not find any loci that affect the genetic susceptibility of steroid-induced ONFH (Table 2).

**Table 2:** Basic information of candidate SNPs in this study

SNP	Gene	Chromosome	Position	Alleles	MAF		HWE	ORs	95% CI		*p*^b^
				A/B	Case	Control	*p*^a^				
rs75267932	MIR17HG	13	91351812	A/G	0.114	0.124	0.411	0.90	0.63	1.30	0.575
rs7336610	MIR17HG	13	91352883	C/T	0.528	0.481	0.129	1.21	0.95	1.52	0.116
rs7318578	MIR17HG	13	91353215	A/C	0.327	0.292	0.389	1.18	0.92	1.51	0.206
rs17735387	MIR17HG	13	91353800	A/G	0.191	0.176	0.358	1.1	0.81	1.48	0.537
rs4143370	MIR155HG	21	25564661	C/G	0.136	0.146	0.597	0.92	0.66	1.28	0.610
rs77218221	MIR155HG	21	25565063	C/T	0.058	0.046	1.000	1.26	0.75	2.10	0.377
rs12482371	MIR155HG	21	25566041	C/T	0.332	0.323	0.104	1.04	0.81	1.33	0.758
rs77699734	MIR155HG	21	25566995	C/G	0.090	0.103	0.636	0.87	0.58	1.29	0.486
rs11911469	MIR155HG	21	25567971	A/C	0.138	0.130	0.545	1.12	0.80	1.57	0.522
rs1893650	MIR155HG	21	25568503	C/T	0.196	0.202	0.491	0.97	0.72	1.29	0.813
rs34904192	MIR155HG	21	25569623	A/G	0.226	0.251	0.288	0.87	0.66	1.15	0.328

*SNP*, single-nucleotide polymorphism; *HWE*, Hardy–Weinberg’s equilibrium; *OR*, odds ratio; *95% CI*, 95% confidence interval; *MAF*, minor allele frequency

^a^*p* values were calculated by exact test

^b^*p* values were calculated by Pearson’s chi-squared test

We further evaluated the association between SNPs and the risk of steroid-induced ONFH in four genetic models (codominant, dominant, recessionary, and additive) by logistic regression analysis adjusted for gender and age. Table 3 shows that SNP rs7318578 was associated with an increased risk of steroid-induced ONFH in the age, gender-adjusted codominant model (OR = 1.01, 95% CI: 0.70–1.46, *p* = 0.039) and recessionary model (OR = 1.78, 95% CI: 1.05–3.00, *p* = 0.032) (Table 3).

**Table 3:** Genotypic model analysis of relationship between SNPs and steroid-induced ONFH

SNP	Model	Genotype	Group = control	Group = case	OR (95% CI)	*p*
rs7318578	Codominant	C/C	47 (9.3%)	27 (13.6%)	1	0.039^*^
		C/A	200 (39.5%)	76 (38.2%)	1.01 (0.70–1.46)	
		A/A	256 (50.6%)	96 (48.2%)	1.79 (1.03–3.09)	
	Dominant	C/C	47 (9.3%)	27 (13.6%)	1	0.427
		C/A–A/A	456 (90.1%)	172 (86.4%)	1.15 (0.82–1.62)	
	Recessive	C/C–C/A	247 (48.8%)	103 (51.8%)	1	0.032^*^
		A/A	256 (50.6%)	96 (48.2%)	1.78 (1.05–3.00)	
	Log-additive	-	-	-	1.23 (0.95–1.58)	0.115

^*^p < 0.05 indicates statistical significance.

The data were stratified according to age, gender, unilateral or bilateral hip joint disease, and course of disease (> 25 months or ≤ 25 months). We further used the *Χ*^2^ test to calculate the difference in allele frequency distribution between the case and the control group in different subgroups, and the results are shown in Table 4. Above age 45, rs77218221 in the *MIR155HG* polymorphism was associated with an increased risk of steroid-induced ONFH (OR = 2.03, 95% CI: 1.02–4.04, *p* = 0.041). In males, rs11911469 in the *MIR155HG* polymorphism was associated with an increased risk of steroid-induced ONFH (OR = 1.53, 95% CI: 1.01–2.30, *p* = 0.0396), whereas rs34904192 reduced the risk of steroid-induced ONFH (OR = 0.67, 95% CI: 0.47–0.96, *p* = 0.0297). In women, rs11911469 in the *MIR155HG* polymorphism reduced the risk of steroid-induced ONFH (OR = 0.50, 95% CI: 0.26–0.94, *p* = 0.0289), and rs4143370 was associated with an increased risk of steroid-induced ONFH (OR = 1.86, 95% CI: 1.04–3.32, *p* = 0.035). In the course of disease, rs77218221 (OR = 2.73,95% CI: 1.16–6.40, *p* = 0.017) and rs34904192 (OR = 1.72, 95% CI: 1.06–2.78, *p* = 0.027) in the *MIR155HG* polymorphism were associated with an increased risk of steroid-induced ONFH (Table 4). The effects of allele and SNP genotypes in different subgroups on steroid-induced ONFH risk were further evaluated, as shown in Table 5. Stratified analysis by age showed that rs77218221 was associated with an increased risk of steroid-induced ONFH in the group older than 45 years of age (OR = 2.75, 95% CI: 1.24–6.08, *p* = 0.013), in the dominant model (OR = 2.65, 95% CI: 1.20–5.82, *p* = 0.016), and in the additive model (OR = 2.39, 95% CI: 1.12–5.08, *p* = 0.023). Rs7318578 was associated with risk reduction of steroid-induced ONFH in patients senior than 45 years of age in the codominant model (OR = 0.82, 95% CI: 0.43–1.55, *p* = 0.042) and the recessive model (OR = 0.83, 95% CI: 1.61–6.92, *p* = 0.022). In the male group, rs34904192 reduced the risk of steroid-induced ONFH in the codominant model (OR = 0.61, 95% CI: 0.39–0.95, *p* = 0.030), the dominant model (OR = 0.61, 95% CI: 0.40–0.94, *p* = 0.024), and the additive model (OR = 0.67, 95% CI: 0.47–0.98, *p* = 0.037). The codominant model of rs11911469 (OR = 0.47, 95% CI: 0.23–0.94, *p* = 0.034), the dominant model (OR = 0.45, 95% CI: 0.22–0.91, *p* = 0.026), and the additive model (OR = 0.45, 95% CI: 0.23–0.88, *p* = 0.021) were associated with reduced risk of steroid-induced ONFH in the female group. In the course group, rs77218221 was associated with an increased risk of steroid-induced ONFH in the dominant model (OR = 2.64, 95% CI: 1.06–6.55, *p* = 0.037), additive model (OR = 2.64, 95% CI: 1.06–6.55, *p* = 0.038) and rs34904192 under the codominant model (OR = 1.22, 95% CI: 0.63–2.35, *p* = 0.043) (Table 5).

**Table 4:** The subgroup information of the MIR17HG gene and MIR155HG gene

Subgroup	SNP	Alleles	MAF		HWE-*p*^a^	ORs	95% CI		*p*^b^
		A/B	Case	Control					
Age,> 45	rs77218221	C/T	0.1	0.052	0.467	2.03	1.02	4.04	**0.041**
Gender, male	rs11911469	A/C	0.164	0.113	1.000	1.53	1.02	2.3	**0.040**
	rs34904192	A/G	0.19	0.259	0.312	0.67	0.47	0.96	**0.030**
Gender, female	rs11911469	A/C	0.1	0.19	0.282	0.50	0.26	0.94	**0.029**
Course, months	rs4143370	C/G	0.187	0.11	0.186	1.86	1.04	3.32	**0.035**
**Case, > 25**	rs77218221	C/T	0.097	0.038	1.000	2.73	1.16	6.40	**0.017**
**Control, ≤ 25**	rs34904192	A/G	0.291	0.193	0.783	1.72	1.06	2.78	**0.027**

*SNP*, single-nucleotide polymorphism; *HWE*, Hardy–Weinberg’s equilibrium; *OR*, odds ratio; *95% CI*, 95% confidence interval; *MAF*, minor allele frequency

^a^*p* values were calculated by exact test

^b^*p* values were calculated by Pearson’s chi-squared test

**Table 5:** The relationship between MIR17HG and MIR155HG gene polymorphism and steroid-induced ONFH subgroup analysis

Subgroup analysis	SNP	Model	Genotype	Control	Case	OR (95% CI)	*p*
Age, > 45	rs77218221	Codominant	C/C	1 (0.4%)	0	1	0.013*
			C/T	22 (9.5%)	14 (20.0%)	2.75 (1.24–6.08)	
			T/T	208 (90.0%)	56 (80.0%)	-	
		Dominant	C/C	1 (0.4%)	0	1	0.016*
			C/T–T/T	230 (99.6%)	70 (100.0%)	2.65 (1.20–5.82)	
		Recessive	C/C–C/T	23 (10.0%)	14 (20.0%)	1	0.999
			T/T	208 (90.0%)	56 (80.0%)	-	
		Log-additive	-	-	-	2.39 (1.12–5.08)	0.023*
Age, > 45	rs7318578	Codominant	C/C	17 (7.4%)	10 (14.3%)	1	0.042*
			C/A	90 (39.0%)	23 (32.9%)	0.82 (0.43–1.55)	
			A/A	121 (52.4%)	37 (52.9%)	2.62 (1.04–6.60)	
		Dominant	C/C	17 (7.4%)	10 (14.3%)	1	0.826
			C/A–A/A	211 (91.3%)	60 (85.7%)	1.07 (0.60–1.91)	
		Recessive	C/C–C/A	107 (46.3%)	33 (47.1%)	1	0.022*
			A/A	121 (52.4%)	37 (52.9%)	2.83 (1.61–6.92)	
		Log-additive	-	-	-	1.30 (0.84–2.01)	0.238
Male	rs34904192	Codominant	A/A	24 (5.7%)	5 (4.3%)	1	0.030*
			A/G	171 (40.4%)	34 (29.3%)	0.61 (0.39–0.95)	
			G/G	228 (53.9%)	77 (66.4%)	0.62 (0.23–1.69)	
		Dominant	A/A	24 (5.7%)	5 (4.3%)	1	0.024*
			A/G–G/G	339 (80.1%)	111 (95.7%)	0.61 (0.40–0.94)	
		Recessive	A/A–A/G	195 (46.1%)	39 (33.6%)	1	0.557
			G/G	228 (53.9%)	77(66.4%)	0.74 (0.27–2.00)	
		Log-additive	-	-	-	0.67 (0.47–0.98)	0.037*
Female	rs11911469	Codominant	C/C	1 (1.2%)	0	1	0.034*
			C/A	29 (34.9%)	17 (20.5%)	0.47 (0.23–0.94)	
			A/A	53 (63.9%)	66 (79.5%)	-	
		Dominant	C/C	1 (1.2%)	0	1	0.026*
			C/A–A/A	82 (98.8%)	83 (100.0%)	0.45 (0.22–0.91)	
		Recessive	C/C–C/A	30 (36.1%)	17 (20.5%)	1	0.999
			A/A	53 (63.9%)	66 (79.5%)	-	
		Log-additive	-	-	-	0.45 (0.23–0.88)	0.021*
Course, months	rs77218221	Codominant	C/C	0	0	1	
**Case, > 25**			C/T	10 (7.6%)	13 (19.4%)	-	
**Control, ≤ 25**			T/T	122 (92.4%)	54 (80.6%)	-	
		Dominant	C/C	0	0	1	0.037*
			C/T–T/T	132 (100.0%)	67 (100.0%)	2.64 (1.06–6.55)	
		Recessive	C/C–C/T	10 (7.6%)	13 (19.4%)	1	-
			T/T	122 (92.4%)	54 (80.6%)	-	
		Log-additive	-	-	-	2.64 (1.06–6.55)	0.038*
Course, months	rs34904192	Codominant	A/A	4 (3.0%)	7 (10.4%)	1	0.043*
**Case, > 25**			A/G	43 (32.6%)	25 (37.3%)	1.22 (0.63–2.35)	
**Control, ≤ 25**			G/G	85 (64.4%)	35 (52.2%)	3.84 (1.04–14.15)	
		Dominant	A/A	4 (3.0%)	7 (10.4%)	1	0.240
			A/G–G/G	128 (97.0%)	60 (89.6%)	1.45 (0.78–2.69)	
		Recessive	A/A–A/G	47 (35.6%)	32 (47.8%)	1	0.052
			G/G	85 (64.4%)	35 (52.2%)	3.56 (0.99–12.82)	
		Log-additive	-	-	-	1.55 (0.95–2.55)	0.082

^*^ *p* < 0.05 indicates statistical significance

**Fig. 1:**
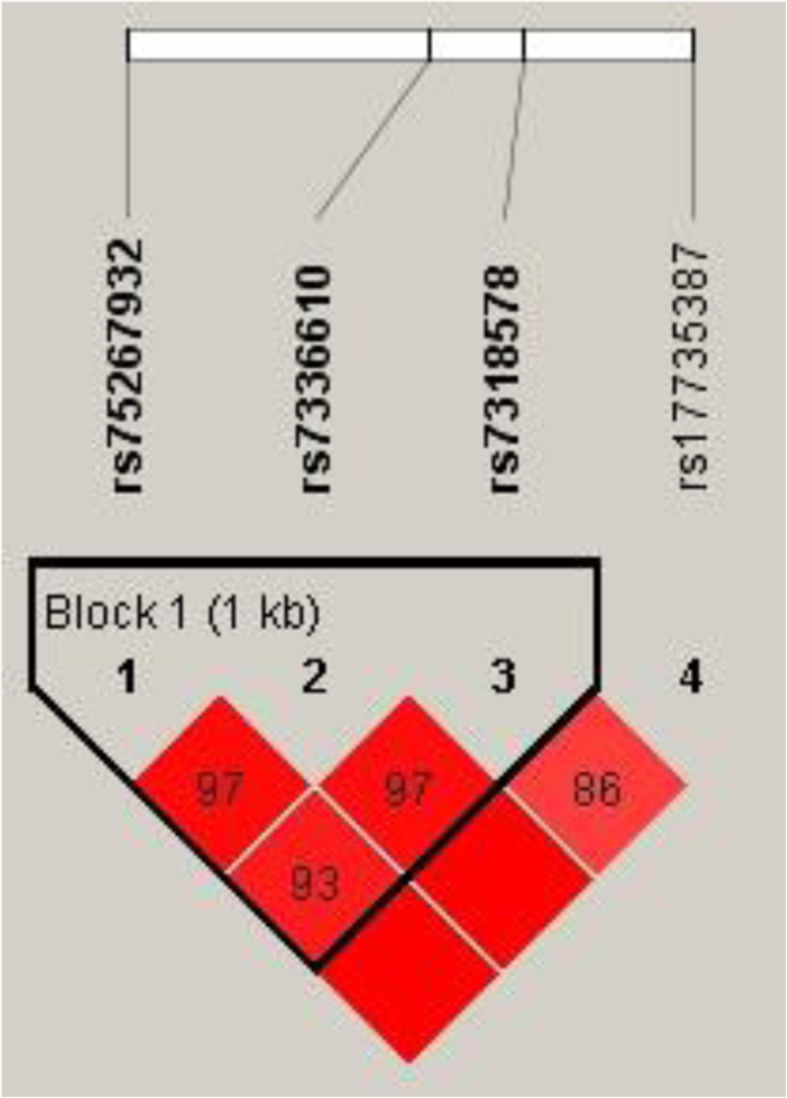
Haplotype block map for the SNPs in MIR17HG gene. The numbers inside the diamonds indicate the D’ for pairwise analyses

**Fig. 2:**
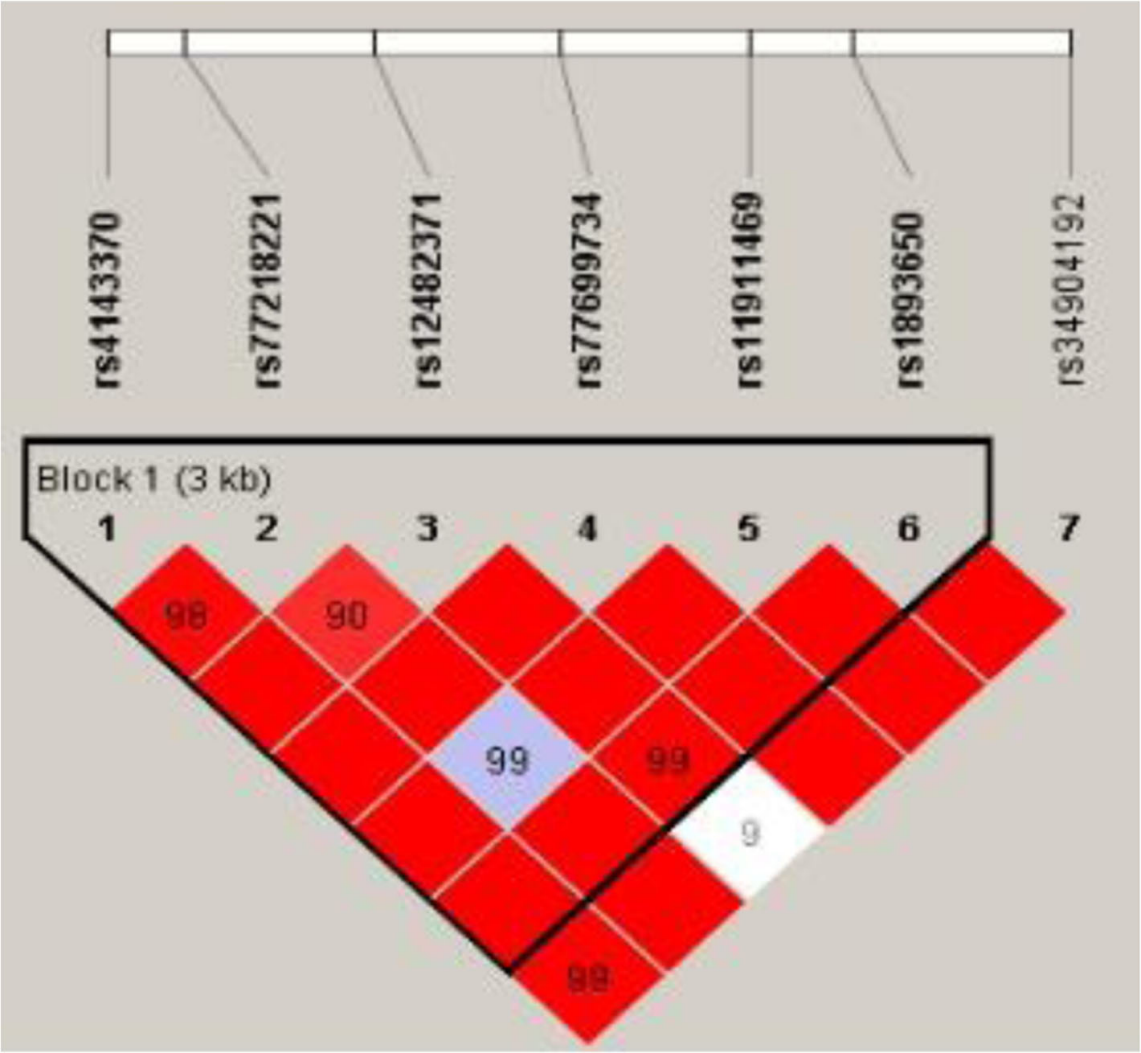
Haplotype block map for the SNPs in MIR155HG gene. The numbers inside the diamonds indicate the D’ for pairwise analyses

Linkage analysis showed that MIR17HG SNPs (rs75267932, rs7336610, and rs7318578) (Fig. 1) and MIR155HG SNPs (rs4143370, rs77218221, rs12482371, rs77699734, rs11911469, and rs189365) (Fig. 2) exhibited significant linkage disequilibrium.

**Fig. 3:**
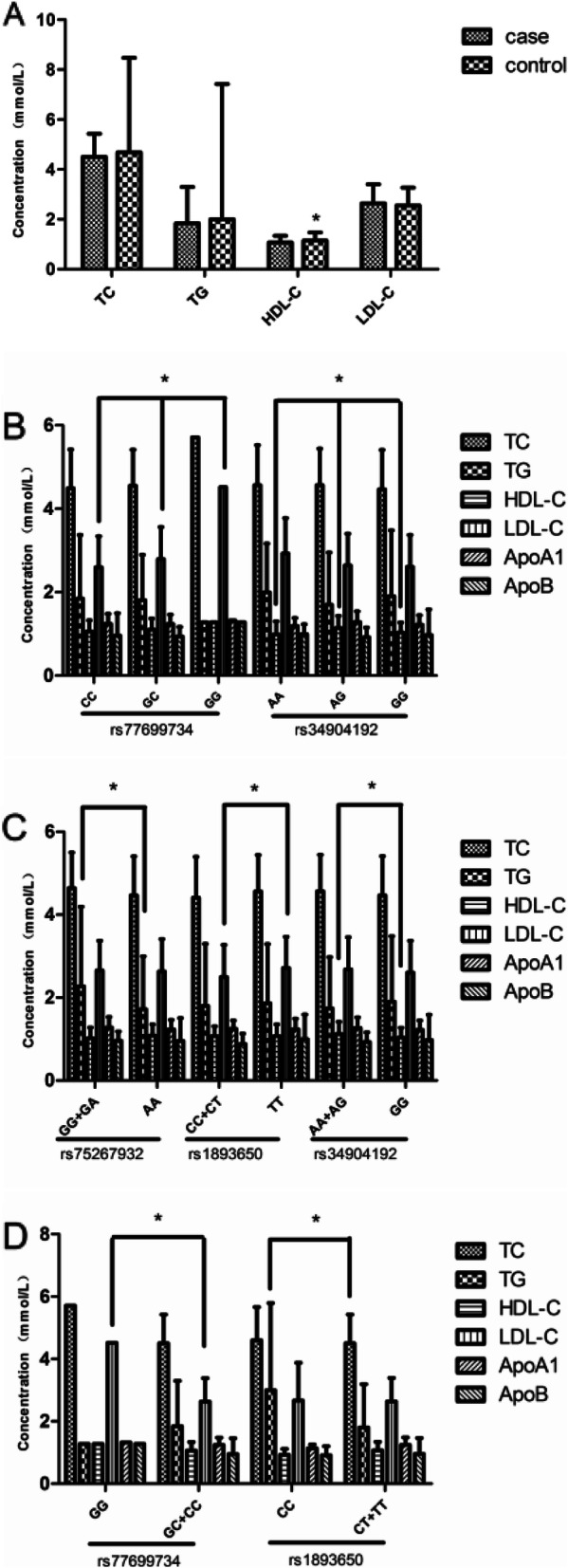
The serum lipid levels between the ONFH and control groups, and the association of MIR17HG and MIR155HG genotypes with the serum lipid levels of ONFH patients. **A** The serum levels of TC, TG, HDL-c, and LDL-c between the ONFH and control groups. *p < 0.005. **B** The association of MIR17HG and MIR155HG genotypes with the serum lipid levels of the ONFH group. Rs77699734, *p < 0.018; rs34904192, *p < 0.022 as indicated. Data are presented as mean ± standard deviation. *ONFH*, osteonecrosis of the femoral head; *TC*, total cholesterol; *TG*, triglyceride; *HDL*, high-density lipoprotein; *LDL*, low-density lipoprotein; *ApoA1*, apolipoprotein A1; *ApoB*, apolipoprotein B. The relationship between MIR17HG and MIR155HG genotypes in the ONFH group and blood lipid levels in ONFH patients. (C) Compare the relationship of blood lipid levels between dominant models of patients' genotypes. TG in Rs75267932, *p<0.027; LDL-C in rs1893650, *p<0.048; HDL-C in rs34904192, *p<0.035. (D) Compare the relationship between blood lipid levels in recessive models of patients' genotypes LDL-C in rs77699734, *p<0.013; TG in rs1893650, *P<0.047; as shown in the figure. Data are expressed as mean ± standard deviation. ONFH, femoral head necrosis; TC, total cholesterol; TG, triglycerides; HDL, high density lipoprotein; LDL, low density lipoprotein; apolipoprotein A1 (ApoA1); apolipoprotein B (apoB)

Analysis of clinical indicators revealed significant differences in *HDL-C* levels between the steroid-induced ONFH group and the control group (*p* = 0.005), as shown in Fig. 3A. The correlation analysis between rs77699734 and rs34904192 genotypes and serum lipid levels showed that there were differences in serum *LDL-C* levels among the three genotypes of rs77699734 GG, GC, and CC (*p* = 0.018), and the serum *LDL-C* level of ONFH patients with the GG genotype was significantly higher than that of patients with the GC or CC genotype. There were relevant differences in serum *HDL-C* levels among ONFH patients with rs34904192 AA, AG, and GG genotypes (*p* = 0.022), as shown in Fig. 3B.

There were significant differences in serum TG between GG + GA and AA (*p* = 0.027) in the rs75267932 governing model, in the *LDL-C* level between CC + CT and TT in the rs1893650 governing model (*p* = 0.048), and in the *HDL-C* level in three genotypes of ONFH patients with AA + AG and GG in the rs34904192 dominant model (*p* = 0.035), as shown in Fig. 3C. In the recessionary model carrying rs77699734, the serum *LDL-C* levels of patients with ONFH between GG and GC + CC were unusual (*p* = 0.013), and the serum *TG* levels of patients with ONFH between CC and CT + TT in the rs1893650 recessive models were distinct (*p* = 0.047), as shown in Fig. 3D.

**Fig. 4:**
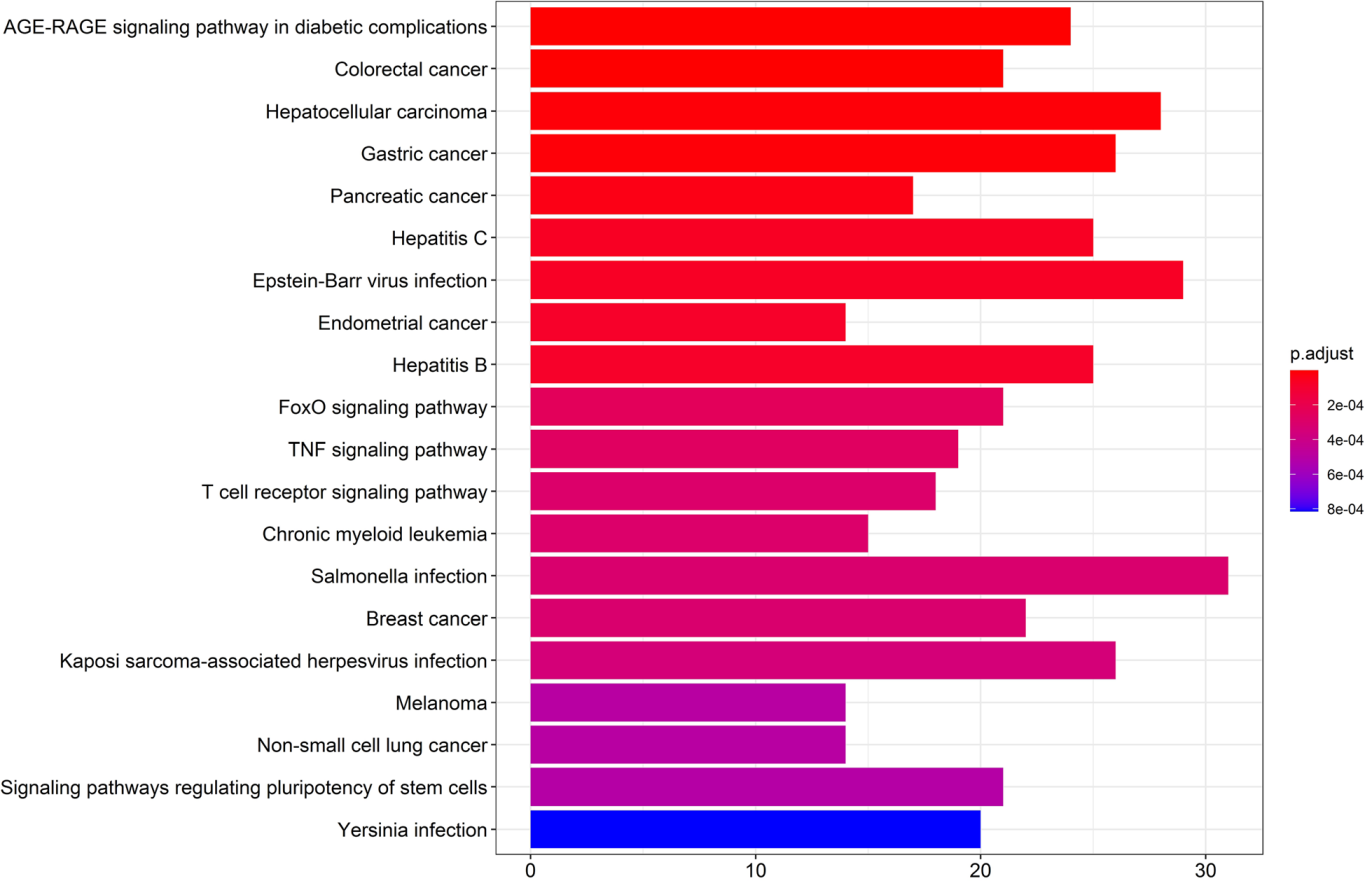
KEGG analysis of downstream genes regulated by MIR155HG

**Fig. 5:**
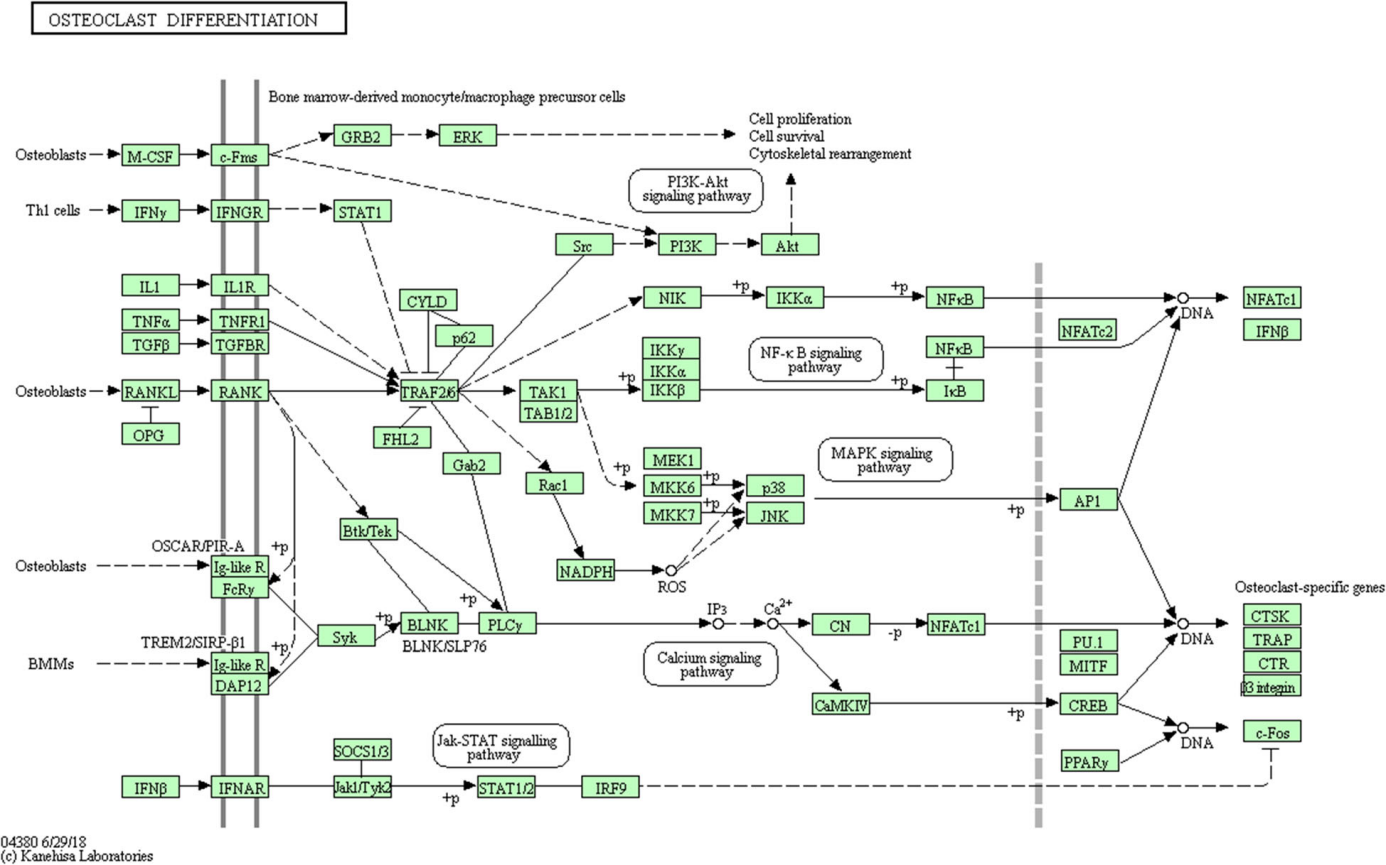
Osteoclast differentiation pathway

In the database (http://www.bio-bigdata.net/LncACTdb/index.html), we found that MIR155HG has a regulatory effect on miR-155-5p. In the (http://mirtarbase.cuhk.edu.cn/) database, we found 919 target genes regulated by miR-155-5p. Through KEGG analysis, we found that the signal pathway that regulates the pluripotency of stem cells and osteoclast differentiation pathway is involved in the occurrence of ONFH has an important role in it. Among them, six miR-155-5p regulated genes (IFNGR1, AKT1, TAB2, MITF, SOCS1, and SOCS3) were found in the osteoclast differentiation pathway, as shown in Figs. 4 and 5.

## Discussion

This study is the first to investigate the selective SNPs in *MIR17HG* and *MIR155HG* and their relationship with steroid-induced ONFH. In the population of northern China, we found that *MIR17HG* SNP (rs7318578) was associated with an increased risk of steroid-induced ONFH. *MIR17HG* SNP (rs7318578) and *MIR155HG* SNPs (rs77218221, rs11911469, rs34904192, and rs4143370) were closely related to distinctive clinical phenotypes of steroid-induced ONFH. *MIR17HG* SNP (rs75267932) and *MIR155HG* SNPs (rs77699734, rs1893650, and rs34904192) were correlated with different lipid indexes.

*MIR17HG* is located on chromosome 13 and has been found to play a prominent role in a variety of human diseases. Studies have confirmed that abnormal expression of *miR-17* has been detected in the local tissues or serum of patients with ONFH [8, 29]. *MiR-17-5p* (located in the *miR-17-92* cluster) delayed the differentiation and proliferation of OBs by downregulating the partial expression of *SMAD7,* leading to the occurrence of nontraumatic ONFH [8, 24]. Studies also found that the *miR-17-92* cluster was downregulated in the OB differentiation of *ES* cells and the bone progenitor cell line Mc3t3-e1 [16]. In the analysis of *miRNA* expression in OBs exposed to apoptotic inducers, it was found that miR-17-92a played a consequential role in protecting estrogen anti-bone loss by regulating Bim expression [6]. These findings suggest that the *miR-17-92* cluster plays a key role in the occurrence and development of steroid-induced ONFH. Previous studies have shown an increased risk of multiple myelomas in patients with the rs7336610 allele of *MIR17HG*, and the three SNPs of *MIR17HG* (rs7336610, rs7318578, and rs17735387) were associated with a risk of colorectal cancer [2, 28]. In this study, rs7318578 was identified as a genetic susceptibility factor for steroid-induced ONFH. To date, only the SNPs (rs7336610, rs7318578, rs17735387, and rs75267932) of *MIR17HG* and the risk of steroid-induced ONFH have been investigated. Therefore, more samples are needed for correlation studies to confirm the results.

OCs are one of the most important cells in maintaining homeostasis, and their excessive bone resorption often leads to bone loss. According to the literature, *miR-155* is involved in the regulation of OCs. In osteoporotic mice, *miR-155* inhibited OC activation by targeting the leptin receptor (*LEPR)* via the *AMPK* pathway [12]. *MiR-155* can target proto-oncogene *SPI1*, microsomia-related transcription factor, and cytokine signal transduction inhibitor protein 1 to inhibit macrophage activation and OC differentiation, thus inhibiting the process of bone resorption [16]. As an intron gene with higher transcription levels, *miRNA* is involved in physiological processes such as development, cell proliferation, differentiation, and metabolism [9]. Research on mesenchymal stem cells of patients with steroid-induced femoral necrosis showed that the overexpression of *miR-155-5p* in their cells can directly inhibit *GSK3B* to promote cell proliferation and bone differentiation [25, 26]. *MiR-155* is the transcription product of the host gene *MIR155HG*, and its expression may be affected by genetic variation in the *MIR155HG* gene. In a study of SNPs in *MIR155HG* and colorectal cancer, rs12482371, rs1893650, rs92888, rs11911469, and rs34904192 increased the risk of colorectal cancer [27]. In our study, the four SNPs of *MIR155HG* (rs77218221, RS11911469, rs34904192, and rs4143370) were associated with a risk of steroid-induced ONFH. Our study suggests that *MIR155HG* may be a new susceptibility gene for steroid-induced ONFH, providing new evidence for the early detection of ONFH.

In a study of the pathogenesis of femoral head necrosis, the lipid metabolism theory is currently the most concerning. Hyperlipidemia can affect the microcirculation of the femoral head from multiple sources (coagulation system, bone fat embolism, and bone microthrombosis), and then lead to femoral head necrosis [6, 14]. With further research, increasing the number of genes have been found to participate in the regulation of lipid metabolism. *SREBP2* can activate target gene transcription and gene expression of cholesterol biosynthesis pathways, which play an important role in lipid homeostasis. Through the study of the relationship between *SREBP2* gene polymorphism and ONFH, it was concluded that *SREBP2* gene polymorphism and function could cause lipid metabolism disorder in ONFH patients [17]. Apolipoprotein is considered to be a sensitive indicator to estimate the disorder of lipid metabolism in the ONFH populatio n[7]. The study found that the -75G/A polymorphism of the *ApoAI* gene may be associated with susceptibility to osteonecrosis in the Chinese population [31]. With the deepening of research, it has been found that miRNA is also involved in the expression of genes regulating lipid transport and metabolism [19, 22]. MiR-122 is the most expressed miRNA in the liver, which regulates the liver cholesterol network either directly by regulating cholesterol synthesis genes or indirectly by another molecular pathway [4]. Studies in primates have shown that inhibition of miR-33a/b can increase plasma HDL-C and decrease VLDL-C and TG [15]. In coronary artery disease, the higher the expression of miR-17 in patients, the higher the levels of TC, LDL-C, and ApoB in vivo. The lower the expression of miR-92a and miR-106b in patients with coronary artery disease, the lower the levels of HDL-C and ApoA-1 in vivo, and these miRNAs may play an independent role [10, 11]. Wan et al. [20] found that miR-17-92 clusters can play an important role in bone, lipid, and glucose metabolism through a variety of signaling pathways. We studied the genotypes and lipid levels of ONFH patients, and found their relationship with clinical phenotype and the development of ONFH. *MIR17HG* and *MIR155HG* may have some effects on lipid metabolism.

## Conclusion

In summary, our study demonstrates that *MIR17HG* SNP (rs7318578) is significantly associated with an increased risk of steroid-induced ONFH in the population of northern China. A *MIR17HG* SNP (rs75267932) and *MIR155HG* SNPs (rs77699734, rs1893650, and rs34904192) were associated with abnormal lipid metabolism. These findings provide evidence for the early screening and prevention of femoral head necrosis. However, more samples from different regions are needed to confirm the results.

## Data Availability

The data that support the findings of this study are available from the corresponding author upon reasonable request.
